# The association of *APOE* genotype with COVID-19 disease severity

**DOI:** 10.1038/s41598-022-17262-4

**Published:** 2022-08-05

**Authors:** Javad Safdari Lord, Javad Soltani Rezaiezadeh, Mir Saeed Yekaninejad, Pantea Izadi

**Affiliations:** 1 Department of Medical Genetics, School of Medicine, Tehran University of Medical Sciences, Tehran, Iran; 2 Department of Epidemiology and Biostatistics, School of Public Health, Tehran University of Medical Sciences, Tehran, Iran

**Keywords:** Genetic association study, Genetic markers

## Abstract

COVID-19 has caused the recent pandemic of respiratory infection, which threatened global health. The severity of the symptoms varies among affected individuals, from asymptotic or mild signs to severe or critical illness. Genetic predisposition explains the variation in disease severity among patients who suffer from severe symptoms without any known background risk factors. The present study was performed to show the association between *APOE* genotype and the severity of COVID-19 disease. The *APOE* genotype of 201 COVID-19 patients (101 patients with asymptomatic to mild form of the disease as the control group and 100 patients with severe to critical illness without any known background risk factors as the case group) were detected via multiplex tetra-primer ARMS-PCR method. Results showed that the e4 allele increased the risk of the COVID-19 infection severity more than five times and the e4/e4 genotype showed a 17-fold increase in the risk of severe disease. In conclusion, since our study design was based on the exclusion of patients with underlying diseases predisposing to severe form of COVID-19 and diseases related to the APOE gene in the study population, our results showed that the e4 genotype is independently associated with the severity of COVID-19 disease. However, further studies are needed to confirm these findings in other nations and to demonstrate the mechanisms behind the role of these alleles in disease severity.

## Introduction

Severe acute respiratory syndrome coronavirus-2 (SARS-CoV-2) is responsible for the recent outbreak of pneumonia that began in early December 2019 in Wuhan, Hubei Province, China^1^. As has been shown so far, patients with COVID-19 show phenotypic variability. Even within a family, affected individuals may show a spectrum of disease presentations: asymptomatic, mild, severe symptoms (leading to hospitalization) and critical condition (requiring intensive care unit and ventilator respiration, and even death)^2,3^. Although there are some known background risk factors for severe form of COVID-19 (such as ages above 65 years, chronic respiratory diseases, diabetes), the reason for disease severity in a subgroup of patients without any known underlying risk factors has not yet been fully understood. Genetic predisposition and the influence of genomic background on the severity of clinical presentation of COVID-19 symptoms are under investigation. Accordingly, many recent studies are looking for genes and single nucleotide polymorphisms (SNPs) that lead to differences in susceptibility to COVID-19 disease^4–6^. One of the effective proteins on the infectivity of the virus was apolipoprotein E (*APOE*) (OMIM number 107741, 19q13.2), according to the UK Biobank study^7^. APOE protein plays a vital role in cholesterol transport, and this protein is the main regulator of plasma lipid levels^8^. Human APOE has three isoforms: e2, e3, and e4. APOE3 (the main form) is associated with normal plasma lipid levels. APOE2 is different from APOE3 by replacing the Arginine158 with Cysteine (rs7412). APOE4 differs from APOE3 by replacing the amino acid Cystein112 with Arginine (rs429358). Based on e2/e3/e4 polymorphisms of the *APOE* gene, there are six different genotypes in human populations^9^. APOE also regulates fat transport and cholesterol homeostasis in the brain^10^, and several studies have shown the association between *APOE* polymorphisms and diseases such as Alzheimer's disease^11^, cardiovascular disease^12^, type 2 diabetes (T2DM)^13^ and brain vascular pathologies^14^, which are comorbidities related to SARS-CoV-2 severity. Based on the biological function of APOE protein and the effect of its variants, it has been suggested that the e4 allele and subsequently the e4/e4 genotype can play an essential role in the severity of Covid-19^15,16^. was The present study aimed to investigate the association between *APOE* genotype and the severity of Covid-19 disease in Iranian patients.

## Result

A total of 101 COVID-19 infected patients with mild symptoms (mean age: 39.90 ± 12.32) participated in this study as the control group. The case group consisted of 100 patients with severe to critical forms of COVID-19 infection (mean age: 46.35 ± 10.24) 12.32). Thirty-four percent of the patients in the control group and forty-five percent in the case group were women.

**Figure 1:**
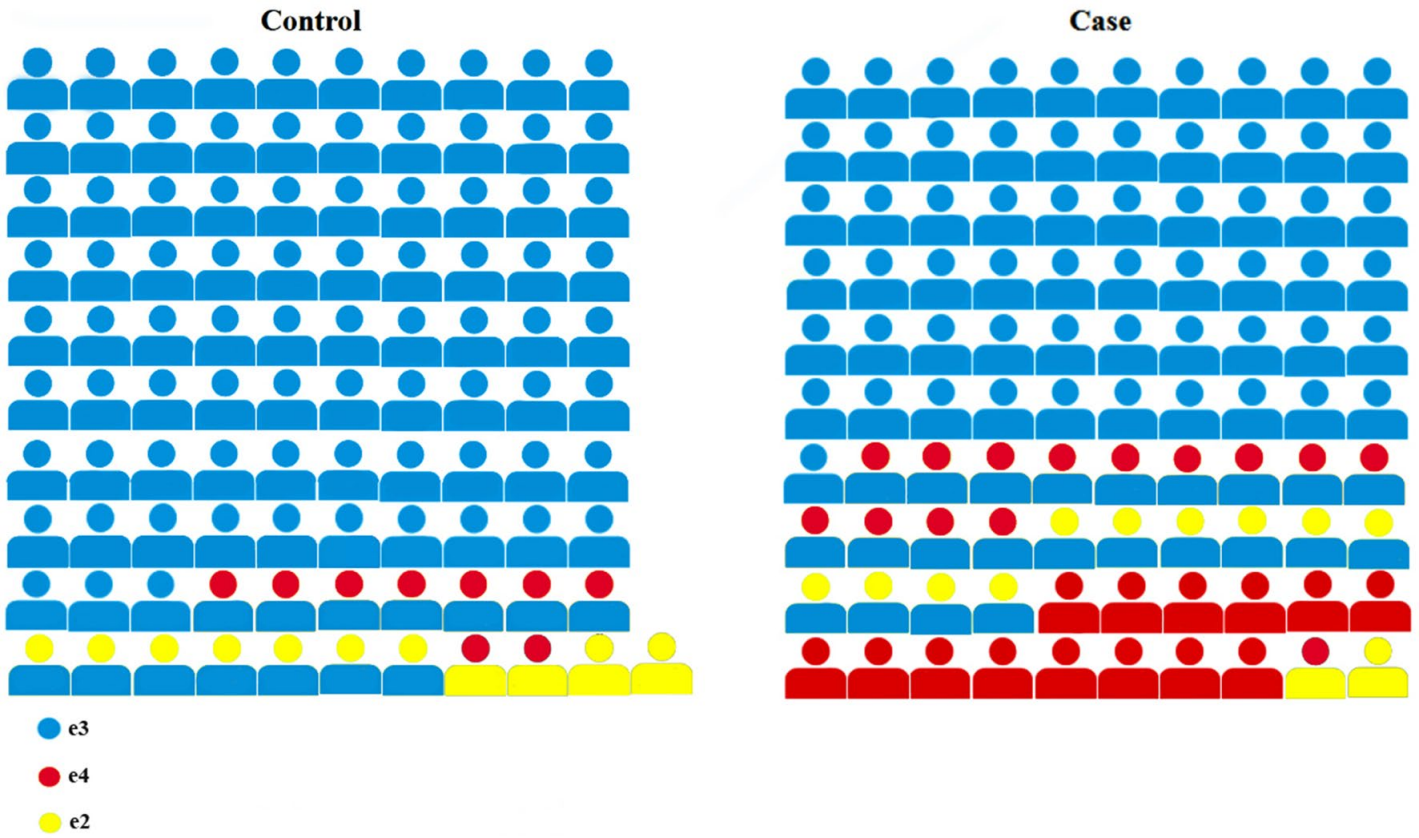
Schematic comparison of genotypes between control and case groups.

*APOE* gene has 6 genotypes based on e2/e3/e4 alleles (e3/e3, e3/e4, e3/e2, e4/e4, e4/e2, and e2/e2). The frequency of genotypes (Table 1) and alleles (Table 2) in the control and case groups were determined. The distribution of genotypes between these two groups is shown graphically in Fig. 1.

**Table 1:** Genotypes frequency of both control and case groups.

		Control group n* = 101 (%)	Case group n* = 100 (%)	P-value	OR (95% CI)*
Genotype	e3/e3	83 (82.2)	61 (61)	9 × 10^–4^	0.33 (0.17–0.64)
	e3/e4	7 (6.9)	13 (13)	1.6 × 10^–1^	2.07 (0.79–5.42)
	e3/e2	7 (6.9)	10 (10)	4.5 × 10^–1^	1.49 (0.54–4.08)
	e4/e4	0 (0)	14 (14)	1 × 10^–4^	17.58 (2.27–135.83)
	e4/e2	2 (2)	1 (1)	1	0.50 (0.04–5.60)
	e2/e2	2 (2)	1 (1)	1	0.50 (0.04–5.60)

*n: sample number, OR: odds ratio, and CI: confidence intervals.

**Table 2:** Allele frequency in control and case groups.

		Control group n* = 101 (%)	Case group n* = 100 (%)	P-value	OR (95% CI) *
Alleles	e3	180 (89.1)	145 (72.5)	1 × 10^–2^	0.69 (0.52–0.92)
	e4	9 (4.5)	42 (21)	2 × 10^–6^	5.09 (2.44–10.61)
	e2	13 (6.4)	13 (6.5)	1	1 (0.45–2.18)

*n: sample number, OR: odds ratio, and CI: confidence intervals.

The statistical analysis of the results showed a significant difference (*P-value* = 1 × 10^–4^) between the distribution of APOE genotype between the control and case groups. All of the six expected genotypes were found in the case group. The e4/e4 genotype did not exist in the control group. The frequency of e3/e3 in the case group compared to the control group showed a significant decrease (*P-value* = 9 × 10^–4^). Also, the e4/e4 genotype frequency in the case group showed a significant (*P-value* = 1 × 10^–4^). The frequency of e3/e4, e3/e2, e4/e2, and e2/e2 genotypes did not show much difference between the case and control groups. Regarding the frequency of alleles, the frequency of the e3 allele (*P-value* = 1 × 10^–2^) decreased in the case group and the e4 allele (*P-value* = 2 × 10^–6^) showed a significant increase in the case group. The e2 allele (*P-value* = 1) showed no difference between the two groups.

## Discussion

Many studies have been conducted to find the causes of differences in the clinical manifestations of COVID-19 and its severity^17–19^. Epidemiological studies have identified several risk factors for the severe form of this viral disease^20,21^. The response to SARS-CoV-2 infection is likely influenced by many host, virus, and environmental factors. Recent studies have focused on host genetic factors to identify possible genetic variants which may be associated with differences in the severity of COVID-19 symptoms^22–24^.

APOE is a multifaceted protein, with three isoforms (e2/e3/e4), associated with several diseases and exhibits isoform-dependent effects. It has been shown that homozygous e4 carriers are particularly vulnerable to medical problems such as cardiovascular disorders, hypercholesterolemia, stroke, Alzheimer's disease, and viral infection^11–13^. Several studies have been performed to find a possible link between *APOE* polymorphism and the severity of COVID-19 symptoms^7,15,25^, and it has recently joined the network of APOE e4-related diseases^26^. Interestingly a recent study involving European ancestry subjects (UK Biobank), found that even by eliminating the role of comorbidities such as type 2 diabetes, dementia, and cardiovascular disease (CAD), the e4 allele can predict the severity of COVID-19 illness^7^. Other studies using UK biobank data have shown that after normalizing the effects of comorbidities such as Alzheimer's disease and CAD, the role of the e4 allele becomes weaker, indicating that these comorbidities add to the role of the e4 allele^27^. An in vitro study examining the association between the e4 allele and the severity of COVID-19 showed that neurons and astrocytes expressing the e4 allele were more susceptible to SARS-CoV-2 infection than those expressing the e3 allele^28^. Another in vitro study found that cells loaded with cholesterol using APOE (cholesterol transport protein) increased endocytic entry of SARS-CoV-2^29^. Therefore, these studies predicted the predisposing role of the e4 allele in COVID-19 clinical outcomes. In the present study, we investigated the association between the *APOE* gene polymorphisms and COVID-19 disease severity in the absence of known *APOE*-related comorbidities in the case group which were risk factors for severe COVID-19. Interestingly, in our study population, the frequency of the e4 allele in the case group was more than 4.5 times higher than in the control group, and this difference was statistically significant (*P-value*: 2 × 10^–6^). Also, our findings showed that the presence of the e4 allele as a risk allele could increase the risk of the disease severity by five times (Table 2). Regarding other *APOE* alleles, the frequency of e2 (*P-value* = 1, OR = 1) was almost constant between the case and control groups and the frequency of e3 (*P-value* = 1 × 10^–2^, OR = 0.6) decreased in the case group. According to these results, the role of e3 and e2 alleles cannot be readily determined, and studies with more samples are needed to determine the role of these two alleles in Covid-19 disease symptoms.

In the present study *APOE* genotype analysis showed that the e4/e4 genotype frequency was significantly different between the case and control groups (*P-value* = 2 × 10^–4^). The odds ratio for the e4/e4 genotype showed that patients with this genotype have a 17-fold increased risk of developing severe COVID-19 disease (Table 1). These findings are consistent with an investigation which showed that the e4 isoform directly induces inflammatory cytokines while the e3 allele regulates these cytokines^30^. Also, it has been shown that having one or two copies of the e4 allele versus two copies of the e3 was associated with an enhanced innate immune response^15^. Thus, the observed genetic association between *APOE* alleles and disease severity is in line with in vitro findings and reiterate that *APOE* e4 allele has a crucial role in COVID-19 severity in patients.

Although our findings about e4 as a risk allele for COVID-19 severity are in concordance with several association studies^7,27,31^, an investigation in a Spanish cohort could not find any association between the e4 allele and COVID-19 severity in patients^32^. It seems that the lower frequency of e4 allele in their studied population along with limited cases with severe form of infection (only 17 patients), can explain part of differences in outcome. Exploring risk alleles in different ethnic groups requires more investigations with a larger sample size.

Finally, for a newly emerging infectious disease such as COVID-19, clarifying host genetic factors for susceptibility to severe form of infection, can be beneficial for designing personalized preventive programs and vaccination. The discovery of genetic susceptibility factors may open up new avenues for developing therapeutic strategies in the future based on the molecular processes related to the clinical consequences of COVID-19 infection.

In conclusion, this study showed an association between the e4 allele and the severity of COVID-19 infection. The e4 allele and e4/e4 genotype can increase the risk of severe COVID-19 by five and seventeen times, respectively. However, studies with a larger sample size in various populations are needed to confirm these findings.

## Methods

### Study population

From September to December 2020, two hundred and one patients with COVID-19 disease participated voluntarily in this study with a positive PCR test from their nasopharyngeal swab samples in Farmanfarmayan Health center (Tehran, Iran). After signing informed consent by each participant, peripheral blood samples were taken from patients in EDTA tubes as anticoagulant. This study has been approved by the ethics committee of the school of medicine of Tehran University of Medical Sciences (Ethics code: IR.TUMS.MEDICINE.REC.1399.785) and has been performed under the Declaration of Helsinki. All experiments and techniques have been performed fallowing relevant guidelines and regulations.

The 101 patients with asymptomatic form or mild symptoms were classified as the control group without age limitation. The 100 patients with severe to critical symptoms (such as a respiratory rate of more than 30 beats per minute, oxygen saturation level (SPO2) less than 90%, lung infiltration more than 50%, and organ failure), who need hospitalization were classified as the case group. All patients with known risk factors for sever form of COVID-19 disease (such as age above 65 years, diabetes, hypertension, heart failure, stroke, cancer chemotherapy or immunodeficiency) were excluded from case group.

### DNA extraction

Total genome DNA was extracted from whole blood samples using the previously described standard salting-out method^33^. The quality and quantity of the purified DNA samples were evaluated by a *Nano-Drop 2000™ spectrophotometer (Thermo Fisher Scientific, USA).* The extracted genomic DNA samples were stored at – 20 °C until genotyping.

### APOE genotyping

Using the multiplex tetra-primer amplification refractory mutation system polymerase chain reaction (Multiplex T-ARMS-PCR) method, the target regions in the *APOE* gene were amplified. In the Multiplex T-ARMS method, two allele-specific primers were used for each SNP, one for the mutant allele (FI-1, RI-2) and the other for the wild-type allele (FI-2, RI-1) and as well as two control primers (RO, FO) was used that amplify the whole fragment. One multiplex PCR reaction was needed for each DNA sample. In multiplex T-ARMS PCR, all six primers (FO, RO, FI-1, RI-1, FI-2, and RI-2) were used in a single reaction tube. Primer sequences have been shown in Table 3. A total volume of 20 µl of the reaction mixture was prepared for each PCR reaction as follows: 10 µl of TEMPase Hot Start 2 × Master Mix A BLUE (Amplicon, Denmark), 0.8 µl genomic DNA sample, 1.6 µl (8%) DMSO (*SinaClon BioScience*, Iran), 0.5 µl (10 pmol/ µl) of each control primers (RO, FO), 1 µl (10 pmol/ µl) of each allele-specific primer and 2.6 µl distilled water. No template control was performed for each experiment by adding 0.8 µl of water instead of sample DNA. The amplification reaction was performed in ABI Veriti thermal cycler machine (*Thermo Fisher Scientific, USA*). The PCR program was carried out with an enzyme activation step at 95 °C for 10 min, followed by 30 amplification cycles (30 s at 95 °C, 30 s at 67 °C, and 30 s at 72 °C) and a final extension at 72 °C for 7 min. The amplification products were separated by electrophoresis on 2% agarose gels (*SinaClon BioScience*, Iran), containing 0.5 mg/L DNA gel stain (*SinaClon BioScience*, Iran) and the image of bands visualized under the UV light and captured by gel documentation system (Syngene, UK).

**Table 3:** Multiplex T-ARMS PCR primers for APOE genotyping.

	Primers	Sequences^a^
Common outer primers	FO	5^´^ ACTGACCCCGGTGGCGGAGGA 3^´^
	RO	5^´^CAGGCGTATCTGCTGGGCCTGCTC 3^´^
Inner primers at codon 112	FI-1	5^´^GGCGCGGACATGGAGGACG**g**G**C** 3^´^
	RI-1	5^´^GCGGTACTGCACCAGGCGGCC**t**C**A** 3^´^
Inner primers at codon 158	FI-2	5^´^CGATGCCGATGACCTGCAGA**c**G**C** 3^´^
	RI-2	5^´^CCCGGCCTGGTACACTGCCAG**t**C**A** 3^´^

^a^The boldface lowercase letters are a deliberate mismatch, and the boldface uppercase letters are an allele-specific mismatch.

The amplification of 115 and 307 base pairs (bp) PCR products indicated the e3/e3 genotype. The presence of 253 bp amplicon showed a variant (C T) which indicated the e2 allele, and the presence of 444 bp amplicon indicated the e4 region variant (TC). The amplification of 514 bp amplicon in each PCR reaction was used as internal control amplicon to demonstrate successful PCR reaction by control primers (Supplementary File)^34^.

### Statistical analysis

Allele and genotype frequencies between case and control groups were compared for, significance using χ^2^ (*Fisher’s exact test)*. Odds ratio (OR) with 95% confidence intervals (CI) were used to describe the strength of association. *SPSS software (version 25)* was used for statistical analysis. All P-values were considered statistically significant for P < 0.05.

## Supplementary Information

Supplementary Information.
