# Supplementary material for: The association of APOE genotype with COVID-19 disease severity
Source: Sci Rep. 2022 Aug 5;12:13483. doi: 10.1038/s41598-022-17262-4 (PMC9356041; doi:10.1038/s41598-022-17262-4)
Supplement: Supplementary file 1 — Supplementary Information. [file 41598_2022_17262_MOESM1_ESM.docx]

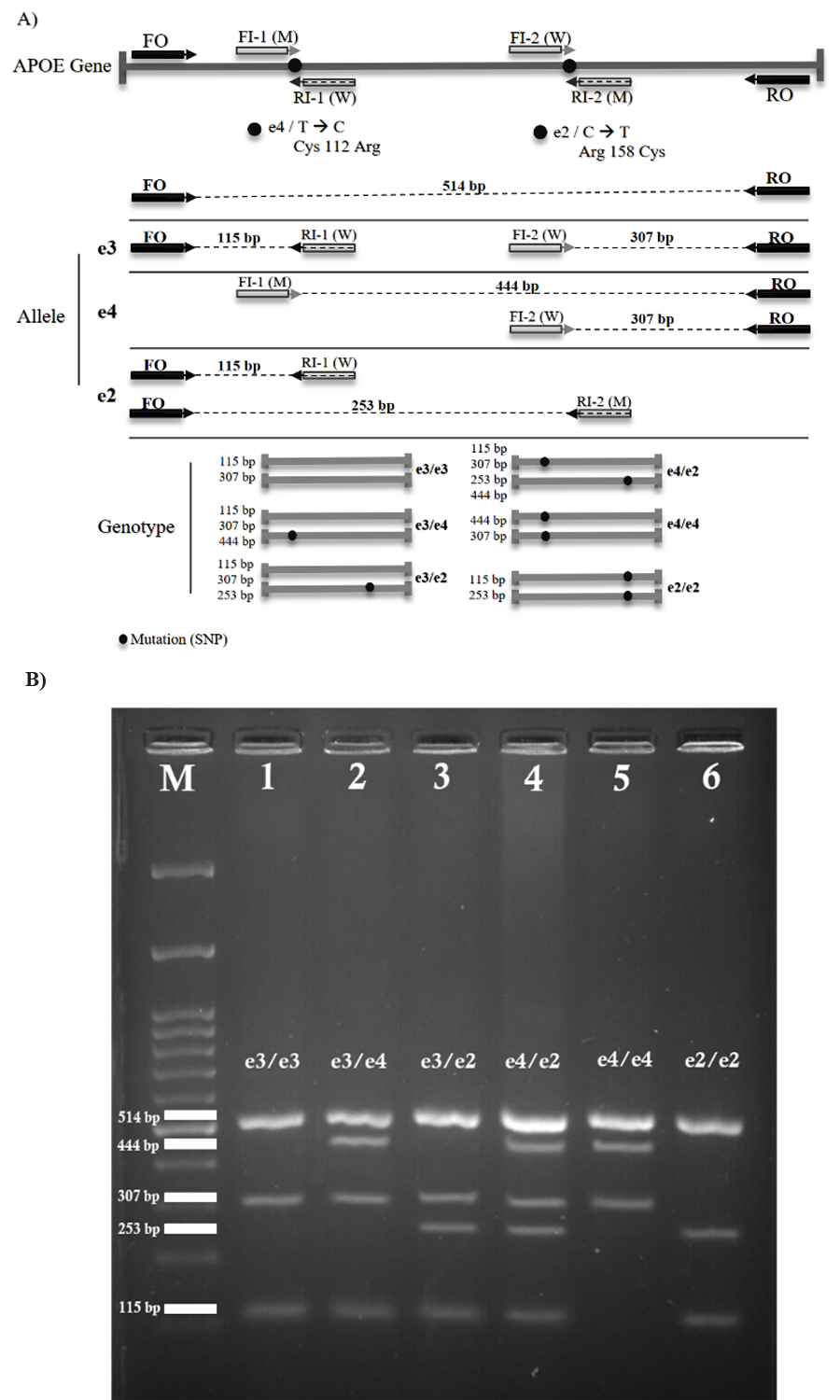


Supplementary figure 1. Multiplex T-ARMS PCR in a single reaction tube. A) Diagram of multiplex T-ARMS PCR for APOE genotyping. Outer primers (FO and RO) amplified 514 bp, and the combinations of each inner primer (FI-1, FI-2, RI-1, and RI-2) and outer primer in two SNP sites produced specific amplicons. The control band (514 bp) is present in all samples. B) Gel electrophoresis of multiplex T-ARMS PCR products. Lane M: 100 bp ladder, Lane 1-6 different genotypes has been detected in different patients.
